# The genus *Deschampsia* and the epithet “ *alpina*”

**DOI:** 10.3897/phytokeys.181.69546

**Published:** 2021-09-03

**Authors:** Jorge O. Chiapella, Zhi-Qing Xue, Josef Greimler

**Affiliations:** 1 Instituto de Investigaciones en Biodiversidad y Medioambiente (INIBIOMA-CONICET-Universidad Nacional del Comahue), Quintral 1250, R8400FRF Bariloche, Río Negro, Argentina Universidad Nacional del Comahue Bariloche Argentina; 2 Department of Botany and Biodiversity Research, Faculty of Life Sciences, University of Vienna, Rennweg 14, 1030 Vienna, Austria University of Vienna Vienna Austria

**Keywords:** *
Aira alpina
*, *
Deschampsia
*, *
Deschampsia alpina
*, *Deschampsiacespitosa* var. *alpina*, *Deschampsiacespitosa* subsp. *alpina*

## Abstract

The epithet “*alpina*” has been recurrently used in the genus *Deschampsia* to name plants located in northern regions of Europe, Asia and North America, as a species (*Deschampsiaalpina* (L.) Roem. & Schult.), but also in infraspecific categories (Deschampsiacespitosasubsp.alpina Tzvel. and Deschampsiacespitosavar.alpina Schur.). The morphological and molecular available evidence suggests the existence of a single species, *Deschampsiacespitosa* (L.) P. Beauv., in which individuals belonging to the same morphological gradient have received different names in different taxonomic categories throughout its wide distribution range. An evaluation of the available names indicates that all uses of the epithet “*alpina*” are illegitimate. A new combination is proposed at the infraspecific level as Deschampsiacespitosasubsp.neoalpina Chiapella, Xue & Greimler.

## Introduction

*Deschampsiacespitosa* (L.) P. Beauv. has a nearly cosmopolitan distribution, being more common in cold temperate regions of the Northern Hemisphere. This extended geographic distribution shows different morphological variants, which have been considered in two alternate ways, either as infraspecific taxa of a widely distributed species (Tzvelev 1976; Clarke 1980; Chiapella 2000; Chiapella and Probatova 2003; Chiapella et al. 2011), or as separate, though related taxa. Authors using the latter approach (separate taxa) include Böcher et al. (1968), Scoggan (1978) and Porsild and Cody (1980). Barkworth (2007) used a mixed approach for North America north of Mexico, accepting three subspecies for *Deschampsiacespitosa* but keeping *D.alpina* at the species level.

Taxa delimitation and nomenclatural problems are common in *Deschampsia* of northern regions of North America, Europe and Asia. The present contribution aims at clarifying the status of the taxon appearing alternatively under the names *Deschampsiaalpina* (L.) Roem. & Schult., Deschampsiacespitosasubsp.alpina Tzvel. and Deschampsiacespitosavar.alpina Schur.

## Morphology

**Figure 1.**
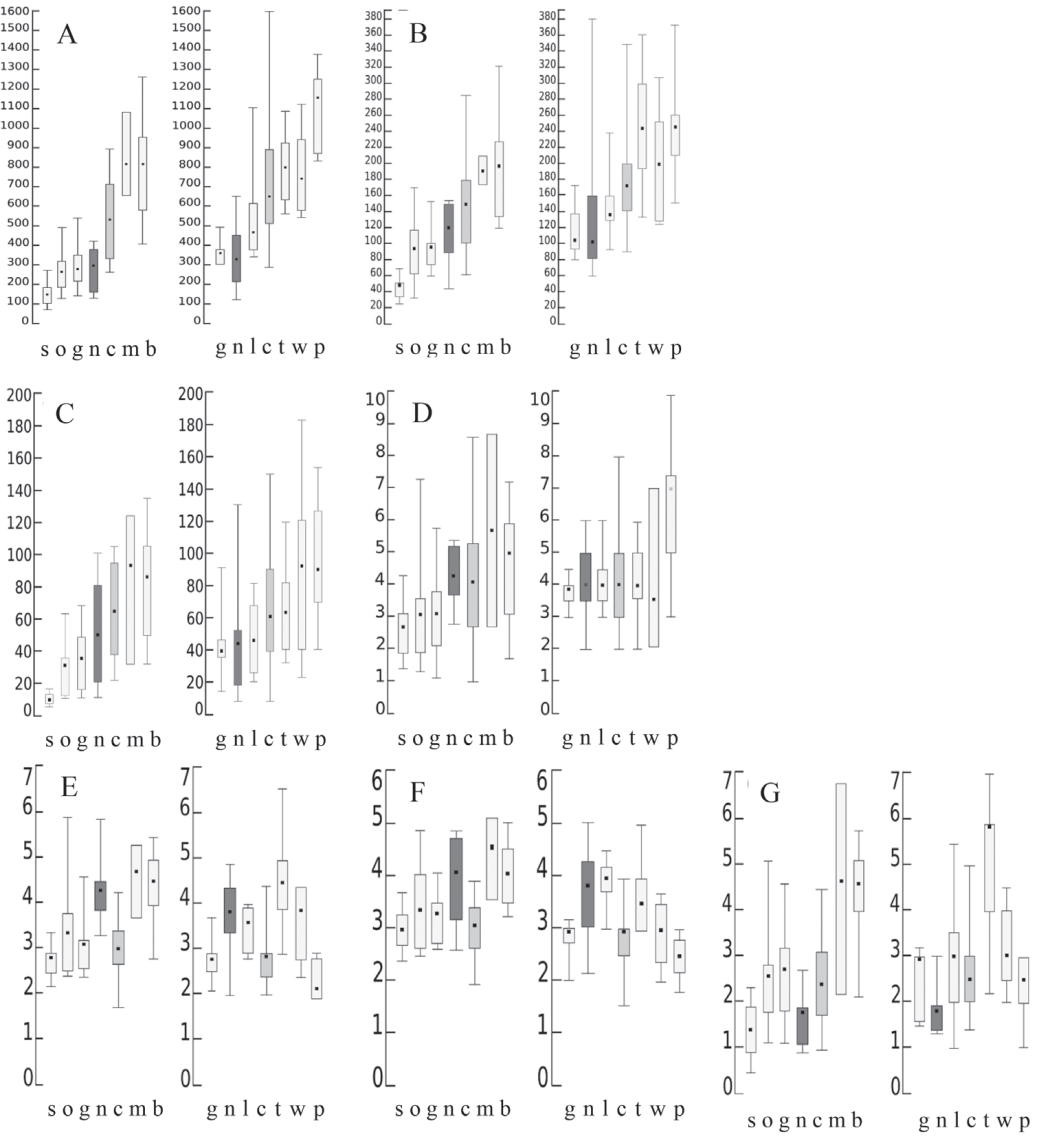
Comparison of selected morphological traits in *Deschampsiacespitosa* s.l. in North America (left panel) and North Central Europe (right panel): plant height **A** panicle length **B** panicle width **C** ligule length **D** lower glume length **E** lemma length **F** awn length **G** taxa codes: Deschampsiacespitosasubsp.septentrionalis (s); D.cespitosasubsp.orientalis (o); D.cespitosasubsp.glauca (g); D.cespitosasubsp.neoalpina (n); D.cespitosasubsp.cespitosa (c); D.cespitosassp.mackenzieana (m); D.cespitosassp.beringensis (b); D.cespitosassp.littoralis (l); D.cespitosasubsp.wibeliana (w); D.cespitosasubsp.parviflora (p). Redrawn from Chiapella (2000) and Chiapella et al. (2011). All in mm.

Morphological quantitative characters in *Deschampsiacespitosa* may vary greatly in short environmental gradients. Plants growing along a 40 m long gradient on tidal soils showed significant differences in plant height and leaf width (Seliskar 1985a, b). Such gradient-like variability at larger scales has been shown in studies on *Deschampsiacespitosa* in Central and Northern Europe (Chiapella 2000) and in North America (Chiapella et al. 2011) (Fig. 1). The environment affects key characters normally used in grass systematics (i.e., plant height, size of panicles, spikelets, glumes and lemmas, length of the awns, etc.).

An additional problem in *Deschampsia* is the development of the sprouting spikelet (glumes, lemmas, paleas), a consequence of the pseudovivipary typical in the taxon named “*alpina*”. These parts elongate beyond the habitual range of *D.cespitosa*, eventually becoming plantlets. Pseudovivipary is an asexual reproductive strategy commonly found in grasses, in which plantlets develop from spikelets and detach from the parental plant after development (Elmqvist and Cox 1996). In *Poa*, pseudovivipary may confer an advantage for growth and dispersal in nutrient-poor habitats (Pierce et al. 2003).

### Cytogenetics

Aiken et al. (2007) provide a summary of available cytogenetical information for *Deschampsiaalpina* (L.) Roem. & Schult. (Table 1). Most of the counts, however, were made in the decades of 1930, 1940 and 1950, with the most recent dates from 1980. Pronounced differences could be due to technical limitations, or to poor chromosome separation in counts of some material (Rothera and Davy 1986: 453). While the many counts cover most of the distribution area of *D.alpina*, their differences might also suggest a possible complex history, with hybridization and chromosome doubling (Levy and Feldman 2002) as probable driving forces of the changes.

**Table 1.** Synopsis of cytogenetic information for *Deschampsiaalpina* (L.) Roem. & Schult. (Source: Aiken et al. 2007).

Count	Distribution	Source
2n = 39, 41, 49.	Spitzbergen, Svalbard Archipelago	Flovik (1938, 1940)
2n = 56.	Northern Europe	Hagerup (1939)
2n = 49.	British Isles	Maude (1939)
2n = 26, 39, 48, 52	Northern Europe	Nygren (1949)
2n = 52.	Greenland	Böcher and Larsen (1950)
2n = 49, 56.	British Isles	Hubbard (1954)
2n = 35–38.	Arctic Russia	Sokolovskaya (1955)
2n = 39, 52.	Iceland	Löve and Löve (1956)
2n = 49, 49 + 2B.	Greenland	Jørgensen et al. (1958)
2n = 26, 38–39.	Arctic Russia	Sokolovskaya and Strelkova (1960)
2n = 39 + 3 – 4ff, 49.	Northern Norway	Engelskjøn and Knaben (1971)
2n = 50.	Bear Island, Svalbard Archipelago	Engelskjøn (1979)
2n = 29 49–52.	Europe, northern Africa	Albers (1980)

The lack of recent cytogenetic studies prevents a comprehensive analysis of the taxon history. However, Aiken et al. (2007) suggest that *Deschampsiaalpina* is either an autopolyploid derivative of Deschampsiacespitosasubsp.cespitosa, or an allopolyploid with one still unknown parent (Elven et al. 2003).

## Taxonomic history

The combination Deschampsiacespitosasubsp.alpina (L.) Tzvel. has been deemed an illegitimate homonym (Aiken et al. 2007) because of the existence of an earlier Deschampsiacespitosavar.alpina Schur. Schur (1859) described this taxon for plants collected during a trip carried out in July-August 1853 to the Carpathian Mountains in Siebenbürgen (present day Romania). The description portrays a high mountain grass growing above the treeline, and was based on the basionym *Airaalpina* L. In the case of Schur’s combination, the Article 11.2 of the Code of Nomenclature (Turland et al. 2018), states that “*a name has no priority outside the rank at which it is published*” thus limiting the priority of the combination of Schur (1859) to the rank of variety. Tzvelev published his combination at the rank of subspecies. The earliest combination of the epithets “*caespitosa*” and “*alpina*” was made as Airacaespitosasubsp.alpina (L.) Hook. in 1870, however in another genus. Furthermore, Article 11.4 (Turland et al. 2018) rules that for infraspecific taxa, the correct name is the combination of the final epithet of the earliest legitimate name of the taxon at the same rank, with the correct name of the genus or species to which it is assigned. Since the correct genus to which this taxon should be assigned is *Deschampsia*, and on account of the reasons mentioned before, the correct category is infraspecific, therefore the combination Deschampsiacespitosasubsp.alpina (L.) Tzvelev would have been used.

However, Article 53.3 (Turland et al. 2018) rules specifies that “*two infraspecific taxa of the same species at different ranks are homonyms if they are not based on the same type*”. In 1869, Ducommun published a textbook on the Swiss flora, including three combinations in *Deschampsiacespitosa* (L.) P. Beauv.: α) D.cespitosa (L.) P. B. var. genuina G.; ß) D.cespitosa (L.) P. B. var. pallida K.; and y) D.cespitosa (L.) P. B. var. alpina G. Explicitly the var. alpina was based on *Airaalpina* Roth non L., based on another type. Therefore, since both names, the one by Schur and the one by Gaudin are based on different types, they are homonyms.

## Discussion

The debates on delimitation of taxa -at specific or infraspecific level- have been recurrent during the 1990’s (Soreng 1991; Luckow 1995; McDade 1995). While now a more sophisticated approach using genomic data and the coalescent is available (Barrett and Freudenstein 2011; Fujita et al. 2012; Leaché et al. 2014), in most cases there is limited molecular information and a lack of basic data on morphology and geographic distribution. The most commonly used strategy has been to differentiate taxa by some character or a combination of characters (Nixon and Wheeler 1990). In cases dealing with infraspecific variation, the preferred approach is to combine morphological variation with geographic allopatry (McDade 1995), which refers to the seminal concept of Du Rietz (1930) – defining subspecies as subtle morphological variants more or less related to defined geographic regions.

The available information for *Deschampsiacespitosa* comprises a set of morphological data for Central Europe (Chiapella 2000) and morphological and molecular data for North America (Chiapella et al. 2011). The morphological data show a gradient-like variability, with recognizable taxa assigned to sections of the gradient (Fig. 1). The molecular information available from the combined *trnK*-*rps*16 spacer and the ITS region yielded 39 haplotypes, two being more abundant and with no obvious relationships between them or to LGM events (Chiapella et al. 2011: 1375). This combined data set was used for maximum parsimony and Bayesian analyses with PAUP* version 4b10 (Swofford 2002) and MrBayes 3 (Ronquist and Huelsenbeck 2003) (for more details, see Chiapella et al. 2011: 1370), and resulted in a single taxon with strong support, *Deschampsiacespitosa*, but with several morphological characters showing a gradient- showing a gradient-like variation corresponding to known infraspecific taxa (Fig. 1).

Since molecular data supports the existence of a single entity, but the morphological data shows variation, the treatment as infraspecific entities seems proper. Consequently, and because all available combinations using the epithet “*alpina*” are illegitimate, a replacement name in reference to Article 41 (Turland et al. 2018) is proposed.

## Nomenclature

### Deschampsia cespitosa subsp. neoalpina

Taxon classificationPlantae

Chiapella, Xue & Greimler.

CFA29299-6798-5881-A3C6-46655CA39A94

≡ Airaalpina L. Sp. Pl. 65. 1753.
≡ Deschampsiaalpina (L.) Roem. & Schult. Syst. Veg. 2: 686. 1817.
≡ Airaalpina Lilj., Utkast Sv. Fl. 49. 1792.
≡ Airaalpinavar.vivipara Parn., Grasses Brit.: 242 t. 109. 1845.
≡ Avenaalpina (L.) Trin., Fund. Agrost.: 157. 1820.
≡ Airacaespitosa subsp. alpina (L.) Hook. f. Student Fl. Brit. Isl. 3: 437. 1870.
≡ Airamajorsubsp.alpina (L.) Syme ex J.E. Sowerby, Engl. Bot. (ed. 3B) 11: 65. 1877.
≡ Deschampsiacespitosa (L.) P.Beauv. var. alpina Vasey in Beal, Grasses N. Amer. 2: 368. 1896.
≡ Deschampsiacespitosasubsp.alpina (L.) Tzvel., in Fed., Fl. Evrop. Chasti SSSR 1: 209. 1974.
= Airaalpina Roth, Tent. Fl. Germ. 2(1): 98. 1789.
= Airalaevigata Sm., Engl. Bot. 30: t. 2102. 1810.
= Deschampsialaevigata (Sm.) Roem. & Schult., Syst. Veg. 2: 687. 1817.
= Deschampsiacespitosavar.alpina Schur, Oesterr. Bot. Z. 9: 326. 1859.
= Deschampsiacespitosavar.alpina Gaudin ex Ducommun, Taschenb. Schweiz. Bot, 861. 1869.
= Deschampsiacespitosa var. alpina (Hoppe) Honda (J. Fac. Sci. Univ. Tokyo (1): 139. 1930.
= Airacaespitosavar.alpina Hoppe, Flora: 166. 1817–1823.
= Deschampsiacespitosavar.alpina (Hoppe) Honda, J. Fac. Sci. Univ. Tokyo, Sect. 3, Bot. a3(1): 139. 1930.
= Airacaespitosavar.alpina Gaudin, Fl. Helv. 1: 323. 1828.
= Deschampsiacespitosavar.alpina Gaudin ex Ducommun, Taschenb. Schweiz. Bot. 861. 1869.
= Airacaespitosavar.alpina Heuff. Verhandlungen der Zoologisch-botanischen Gesellschaft in Wien 8: 228. 1858.
= Airavivipara Steud., Syn. Pl. Glumac. 1: 222. 1854.
= Airaalpinavar.vivipara (Steud.) Lange, Consp. Fl. Groenland. 3: 163. 1880.

#### Type.

Sweden, Torne Lappmark, Mt. Njuolja, 25.07.1950, leg. N.D.Simpson 50133 (BM), neotype selected by Cope in Cafferty et al., Taxon 49: 293. 2000.

## Supplementary Material

XML Treatment for Deschampsia cespitosa subsp. neoalpina
